# Neuroprotective Effects of Quercetin on Ischemic Stroke: A Literature Review

**DOI:** 10.3389/fphar.2022.854249

**Published:** 2022-05-18

**Authors:** Leilei Zhang, Jingying Ma, Fan Yang, Sishi Li, Wangran Ma, Xiang Chang, Lin Yang

**Affiliations:** ^1^ Xi’an Hospital of Traditional Chinese Medicine, Xi’an, China; ^2^ Shaanxi University of Chinese Medicine, Xianyang, China; ^3^ Guang’anmen Hospital, Chinese Academy of Chinese Medical Sciences, Beijing, China

**Keywords:** quercetin, ischemic stroke, inflammatory thrombus, neuroprotection, immune cell activation, mechanism

## Abstract

Ischemic stroke (IS) is characterized by high recurrence and disability; however, its therapies are very limited. As one of the effective methods of treating acute attacks of IS, intravenous thrombolysis has a clear time window. Quercetin, a flavonoid widely found in vegetables and fruits, inhibits immune cells from secreting inflammatory cytokines, thereby reducing platelet aggregation and limiting inflammatory thrombosis. In pre-clinical studies, it has been shown to exhibit neuroprotective effects in patients with ischemic brain injury. However, its specific mechanism of action remains unknown. Therefore, this review aims to use published data to elucidate the potential value of quercetin in patients with ischemic brain injury. This article also reviews the plant sources, pharmacological effects, and metabolic processes of quercetin *in vivo*, thus focusing on its mechanism in inhibiting immune cell activation and inflammatory thrombosis as well as promoting neuroprotection against ischemic brain injury.

## 1 Introduction

Quercetin, which is present in many plants, has become a nutraceutical because of its significant antioxidant and anti-inflammatory activities (Shen et al., 2021; Zou et al., 2021), especially with its ability to scavenge free radicals (Anand David et al., 2016). Clinical studies have shown that quercetin has certain therapeutic effects on cardiovascular diseases (Dehghani et al., 2021), metabolic syndrome (Leyva-Soto et al., 2021), COVID-19 (Di Pierro et al., 2021), and central nervous system diseases.

Ischemic stroke (IS) is caused by hypoxic necrosis of brain tissue due to impaired blood supply to the brain, thereby leading to ischemia. It is the third leading cause of death worldwide (GBD, 2021). In 2017, there were 80.5 (UI78.9-82.6) deaths per 100,000 people, of which 45% were related to IS (Avan et al., 2019); IS accounted for 62.4% of stroke events in 2019 (GBD, 2021). Various risk factors, such as hypertension, diabetes, high body mass index, and smoking, determine the prevalence of IS and its complications; the antioxidant and inflammatory balance mechanisms in the body are severely damaged, thus causing an increase in neuronal reactive oxygen species (ROS), dysfunction, calcium excess, and oxidative stress (Lo et al., 2003).

**FIGURE 1:**
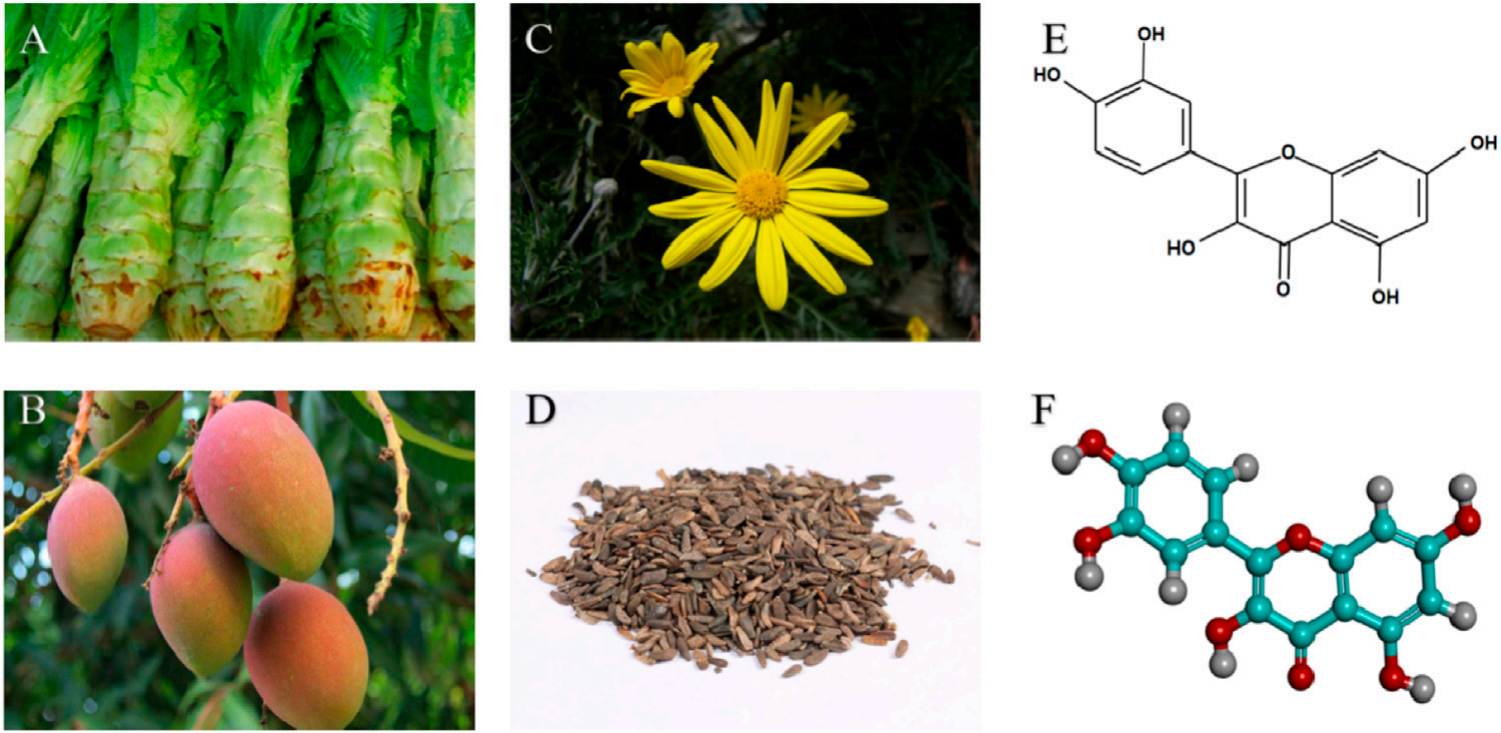
Natural sources and structures of quercetin: **(A)** Lettuce Rootstock; **(B)** Mango fruit; **(C)** Arnica flowering plants; **(D)** Dried ripe fruit of burdock; **(E)** Chemical structure of quercetin; **(F)** 3D structure of quercetin (Visualized by DiscoveryStudio 2016).

Vitamins, carotenoids, and quercetin, which are natural antioxidants, are effectively used to prevent IS. Their mechanism may be related to the synergistic effects of vitamins and antioxidants (Ratnam et al., 2006). The molecular structure of quercetin is C15H10O7 (Magar and Sohng, 2020), which means that there is one -OH at each of the 3, 3′, 5, 7, and 4′ positions (Rice-Evans et al., 1997), and is present in a variety of plants and fruits (Figure 1). Quercetin is a polyphenol belonging to the flavonoid family (Brüll et al., 2015). Inflammatory thrombus formation exacerbates nerve damage in IS (De Meyer et al., 2022). Clinical studies have found that oral quercetin can reduce collagen-stimulated platelet tyrosine phosphorylation and thus inhibit platelet aggregation (Hubbard et al., 2004). Quercetin pre-treatment also reduces lipopolysaccharide-induced neutrophil IL-6 secretion (Liu J. et al., 2005). This process slows the formation of inflammatory thrombi, thus reducing the occurrence of IS.

Recent studies have demonstrated the neuroprotective properties of quercetin in *in vivo* and *in vitro* IS models (Yang et al., 2021). Therefore, this article is the first to describe the source and physicochemical properties of quercetin as well as the pathogenesis of ischemic brain injury. The therapeutic potential of quercetin in ischemic brain injury has been highlighted, including its role in limiting the secretion of inflammatory factors by various immune cells, thereby inhibiting inflammatory thrombosis, oxidative stress, apoptosis, autophagy.

## 2 Source and Physicochemical Properties of Quercetin

The term, “*quercetin*,” has been used since the mid-18th century and is derived from the Latin word, “*quercetum*” (Jaimand et al., 2012). Quercetin is highly lipophilic and has poor water solubility, rapid metabolism, short half-life, and low bioavailability (Mukhopadhyay and Prajapati, 2015). Meanwhile, it is a unique polyphenol found in large quantities in various leafy vegetables, fruits, and herbs, such as apples, berries, long-leaf berry cilantro, cumin, lingonberry, lingonberry, wild grapes, and onions (Yang D. et al., 2020; Sharifi-Rad et al., 2021). According to previous research, quercetin has more than seven biological features, including neuroprotection, anti-allergy, anti-oxidation, anti-inflammation, immune regulation, anti-microbial, and anti-tumor properties (Bjeldanes and Chang, 1977; Dajas, 2012; Oboh et al., 2016; Darband et al., 2018; Dhiman et al., 2019; Huang et al., 2020; Shabbir et al., 2021). However, some studies have indicated that quercetin can induce mutations and promote mutagenesis (Bjeldanes and Chang, 1977). Conversely, Sumi et al. (2013) found that quercetin glucoside promoted angiogenesis after ischemia, but did not promote tumor growth.

In addition, the effectiveness of quercetin depends mainly on the plant source, dose, and chemical properties after processing (Najda et al., 2019). It can also be combined with salivary proteins to form soluble protein-quercetin binary aggregates. It is generated in the small intestines and is directly absorbed by the sodium-dependent glucose transporter-1 in the cecum and colon (Manach et al., 2004). Quercetin is also absorbed by intestinal epithelial cells, thus entering the liver through lipophilic dispersion and undergoing metabolism (Shen et al., 2021). In humans, quercetin has very low bioavailability and is highly unstable (0–50%), with a half-life of 1–2 h in the body after ingesting quercetin-rich foods or supplements (Graefe et al., 1999). Furthermore, quercetin poorly crosses the blood-brain barrier (BBB) (Oliveira et al., 2021). After dietary absorption, quercetin is digested and metabolized extremely quickly; therefore, its pharmacological effects are concentrated on *in vitro* studies rather than *in vivo* (Williams et al., 2004; Barnes et al., 2011). Therefore, various approaches have been attempted to improve the bioavailability of quercetin in the brain, such as enzyme modification or nano-encapsulation (Pateiro et al., 2021). Simultaneously, nanotechnology and targeted vectors are solutions to overcome the shortcomings of quercetin, such as low bioavailability and poor BBB passage (Naseri et al., 2015). The bioavailability of quercetin is 50 times higher than that of standard quercetin products after being packaged into nanocapsules (Riva et al., 2019). Alternatively, it changes the basic structure of quercetin to improve its pharmacokinetic and neuroprotective abilities (Chen et al., 2005). Table 1 summarises the sources of quercetin.

**TABLE 1:** Sources of quercetin.

Scientific Name	Walnut	Active Portions	Family	References
*Davidia involucrata* Baill	Dove tree	Fruits and seeds	Nyssaceae	Girardelo et al. (2020)
*Mangifera indica* L.	Mango	Fruits and Kernels	Anacardiaceae	Mwaurah et al. (2020)
*Arctium lappa* L.	Great Burdock Achene	Fruits and roots	Asteraceae	Moro and T P S Clerici, (2021)
*Punica granatum* L.	Pomegranate	Leaves and fruits	Lythraceae	Rojas-Garbanzo et al. (2021)
*Theobroma speciosum*	Theobroma	Shells and beans	Sterculiaceae	Mar et al. (2021)
*Allium cepa* L.	Onion	Bulbs	Liliaceae	Fredotović et al. (2021)
*Capsicum annuum* L	Sweet Pepper	fruits	Solanaceae	Guevara et al. (2021)
*Syringa vulgaris* L.	Lilac	Flowers and leaves	Oleaceae	Hanganu et al. (2021)
*Sorbus aucuparia* L.	mountain-ash	Fruits	Rosaceae	Rutkowska et al. (2021)
*Gracilaria*	Seaweed	Fruits	Gracilariaceae	Pourakbar et al. (2021)
*Musa nana* Lour	Banana	Skins and fruits	Musaceae	Bashmil et al. (2021)
*Lactuca sativa* L.	Lettuce	Leaves	Asteraceae	Assefa et al. (2021)
*Abies alba* Mill	Silver fir	Leaves	Pinaceae	Vek et al. (2021)
*Juglans regia* L.	Walnut	Nuts	Juglandaceae	Kalogiouri and Samanidou, (2021)
*Malus pumila* Mill	apple	Peels and fruits	Rosaceae	Yousefi-Manesh et al. (2021)
*Arnica montana* L.	*A. Montana*	Flowers and roots	Asteraceae	Nieto-Trujillo et al. (2021)
*Paronychia argentea* L	*P. argentea*	Leaves and Herbs	Caryophyllaceae	Abdelkhalek et al. (2021)

## 3 Pathogenesis of Ischemic Stroke

### 3.1 Inflammatory Thrombus

In the pathophysiological process of IS, inflammatory thrombi lead to cerebral vascular occlusion, inflammatory response, and severe nerve damage after ischemic events (De Meyer et al., 2022). Early platelet adhesion and activation are key factors for the development of IS inflammatory thrombosis. The main receptors that mediate platelet adhesion are glycoprotein (GP) VI and integrin α2β1, both of which bind to the GPIbα subunit of collagen and the GPIB-IX-V complex, which interact with the von Willebrand factor (vWF) (Poulter et al., 2017; Constantinescu-Bercu et al., 2022; Feitsma et al., 2022). After endothelial injury, vWF interacts with GPIbα, thus causing platelets to decelerate on the fixed vWF (Constantinescu-Bercu et al., 2022; Kanaji et al., 2022) and thereby contributing to platelet aggregation. The use of GPIbα-vWF inhibitors restores vascular patency by specifically breaking down the outer layer of the occlusive thrombus (Le Behot et al., 2014). Subsequently, platelet activation induces a conformational change in the GPIIb/IIIa surface receptor and its affinity to fibrinogen and vWF, thus promoting platelet aggregation (O'Brien and Salmon, 1990).

In addition, vWF was found in different samples of thrombus extracted from IS patients; the thrombus contained 20.3% ± 10.1% vWF on average (Denorme et al., 2016). In a middle cerebral artery occlusion (MCAO) rat model, cerebral infarct size and fibrinogen deposition were significantly increased in platelet-only vWF chimeric rats (Verhenne et al., 2015). Interestingly, vWF can be cleaved by metalloprotease ADAMTS13, a disintegrin and metalloproteinase with a thrombospondin type 1 motif member 13. ADAMTS13 effectively dissolves anti-tissue-plasminogen activator (t-PA) thrombus within 5–60 min of MCAO occlusion (Denorme et al., 2016). Furthermore, caADAMTS13, a ADAMTS13 variant, significantly reduced residual vWF, fibrin, and platelet aggregation as well as neutrophil recruitment in the middle cerebral artery (MCA) (South et al., 2022).

However, thrombosis not only involves simple platelet aggregation, but also includes leukocyte-platelet complexation (Li et al., 2015; Pircher et al., 2019; Schrottmaier et al., 2020). This may be because basic diseases, such as hyperlipidemia and hyperglycemia, stimulate hematopoietic cells in the bone marrow to produce a large number of white blood cells in the circulating blood (Stumvoll et al., 2005; Zhou et al., 2016; Vekic et al., 2019). Neutrophils are closely related to thrombosis in IS patients with COVID-19 (Genchi et al., 2022). Neutrophils account for the majority of leukocytes in IS thrombi, followed by macrophages and T cells (Heo et al., 2020). This difference is partly due to their proportion in the circulating blood under physiological conditions; however, IS is also related to the level of activation of various white blood cells. Thrombus formation is a series of complex events that occur sequentially in the vascular system, including endothelial activation, neutrophil extracellular trap (NET) formation, vWF secretion, blood cell adhesion, aggregation, and activation (Yang J. et al., 2020). Genetic deletion of PKM2 in bone marrow hematopoietic cells reduces NET after cerebral ischemia/reperfusion, which further reduces fibrinogen, platelet deposition, and inflammatory cytokines in the brain (Dhanesha et al., 2022). Similarly, rats lacking CD84 on their platelets or T cells showed reduced cerebral thrombosis and milder nerve damage after MCAO (Schuhmann et al., 2020). In contrast, endothelial CD69 deficiency increases fibrinogen and vWF levels in ischemic tissue and exacerbates nerve injury (Brait et al., 2019). Thus, inhibition of inflammatory thrombi formation is one of the goals of IS prevention.

### 3.2 Immune Activation

Activation of immune cells, including neutrophils, T cells, and microglia, is involved in brain tissue repair after IS (Ma et al., 2017). Subsequently, neutrophils are attracted along a concentration gradient of chemokines in areas of ischemia to release pro-inflammatory factors, ROS, proteases, and matrix metalloproteinases (MMPs) (Wang et al., 2007), thus leading to the disruption of the BBB and exacerbation of neurological damage (Rosell et al., 2008). Similarly, in the acute phase of IS, Th1 and Th17 cells degrade tight junction proteins (TJ) by secreting IFN-γ, IL-17, and IL-21, thereby further disrupting the integrity of the BBB (Gelderblom et al., 2012; Clarkson et al., 2014). T cells and their isoforms have also been associated with repair and functional improvement in late brain injury (Liesz et al., 2009). During ongoing inflammation, activated M1 microglia phagocytose astrocyte ends and disrupt the integrity of the BBB by secreting various vascular proteins (Wang et al., 2018; Haruwaka et al., 2019).

### 3.3 Oxidative Stress

Oxidative stress and mitochondrial dysfunction are important factors for the development of cerebral ischemic injury (Lo et al., 2003). Mitochondria are central to ROS production and cell death (Lo et al., 2003). Cerebral ischemia induces a cascade of excessive ROS production. Excess ROS leads to lipid peroxidation (LPO), exacerbates oxidative damage to proteins and nucleic acids, and contributes to neuronal apoptosis and BBB destruction (Allen and Bayraktutan, 2009; Kleinschnitz et al., 2010; Casas et al., 2017; Sun et al., 2018). Immune activation and oxidative stress also contribute to programmed neuronal death in the ischemic zone.

### 3.4 Procedural Death

Both hypoxia and ischemia induce autophagy. Shortly thereafter, autophagic vesicles accumulate extensively in the brain tissue (Tuo et al., 2021). Mitochondrial autophagy facilitates the maintenance of cellular homeostasis under mild ischemia or hypoxia. In contrast, sustained ischemia-reperfusion (I/R) results in prolonged autophagy, thus promoting neuronal cell damage or even death (Zhang et al., 2013). Similarly, neuronal apoptosis is the main mechanism through which I/R injury induces cell death. The balance between anti-apoptotic Bcl-2 and pro-apoptotic Bax protein expression is critical for the regulation of apoptosis (Culmsee and Plesnila, 2006). ROS production and mitochondria-dependent apoptosis play an important role in neuronal death following I/R injury (Jordan et al., 2011; Wang et al., 2014). After IS, a series of molecular events induced by oxidative stress overlap with iron sagging/oxidation processes; these have common molecular targets, such as LPO and glutathione (GSH) depletion (Seiler et al., 2008; Choi et al., 2013; Yang et al., 2014). Iron death is dependent on excessive iron accumulation, with the core process being LPO (Cao and Dixon, 2016). In a rat model of MCAO, GSH inhibited iron death by driving glutathione peroxidase 4 (GPx4) expression, thereby protecting neurons and reducing core ischemic areas (Karuppagounder et al., 2016; Alim et al., 2019).

## 4 Pharmacological Effects of Quercetin on Ischemic Stroke

### 4.1 Inhibition of Immune Cell Recruitment

**FIGURE 2:**
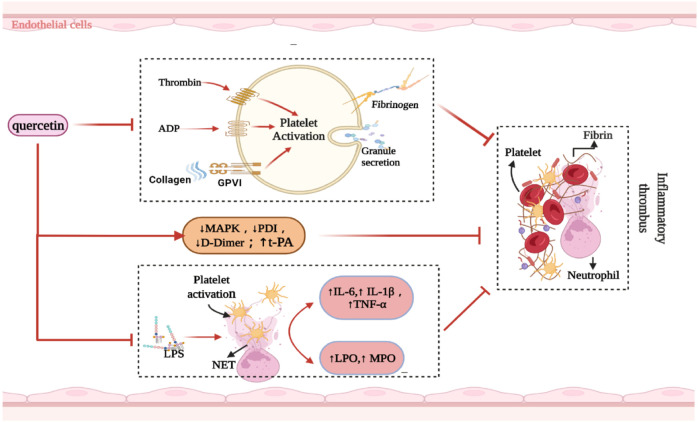
Summarized the mechanism of quercetin inhibiting inflammatory thrombosis. Abbreviations: ↑, increase; ↓, decrease; ADP, Adenosine diphosphate; PDI, Protein disulfide isomerase; NET, neutrophil extracellular trap; LPO, lipid peroxidation; MPO, myeloperoxidase; t-PA, anti-tissue-plasminogen activator; MAPK, mitogen-activated protein kinase; LPS, lipopolysaccharide; GPVI, glycoprotein VI; IL, interleukin; TNF-α, tumor necrosis factor-alfa.

The activation of peripheral immune cells promotes platelet aggregation. Pre-treatment of activated T cells with quercetin blocks IL-12-induced JAK-STAT tyrosine phosphorylation, thereby reducing T cell proliferation and Th1 differentiation (Muthian and Bright, 2004). Quercetin has a similar effect on neutrophils. NETs are closely associated with inflammatory thromboses. Quercetin does not directly affect NET formation, but inhibits it in peripheral blood polykaryotic cells by downregulating TNF-α production in lipopolysaccharide (LPS) peripheral blood monocytes (Yuan et al., 2020; Jo et al., 2021). During inflammation, LPS delays the spontaneous apoptosis of neutrophils, while quercetin accelerates this process (Liu J. J. et al., 2005; Yuan et al., 2020). This is associated with a reduction in the expression of inflammatory cytokines, activation of PKCa, and enhancement of CD95-mediated apoptosis in neutrophils (Russo et al., 2003). Quercetin effectively protects LDL from neutrophil-mediated modification at physiological concentrations (1 µM) and inhibits myeloperoxidase (MPO) oxidative damage (Loke et al., 2008) (Figure 2). Subsequently, quercetin downregulates the TLR-NF-κB signaling pathway, reduces the activities of COX, 5-LOx, NOS, MPO, and CRP, inhibits ldL-induced adhesion molecule expression, and ameliorates endothelial dysfunction in atherosclerosis (Bhaskar et al., 2016). Quercetin also reduces the activity of neutrophil MPO and inhibits the production of HOCl, a powerful oxidant, to protect endothelial cells from oxidative damage (Lu et al., 2018). In contrast, Suri et al. (2008) found that quercetin did not exert excessive influence on neutrophils, but only reduced the calcium response induced by N-formyl-methionyl-leucyl-phenylalanine (fMLP).

### 4.2 Inhibition of Thrombosis

The key factor for the occurrence of IS is the formation of an inflammatory thrombus; thrombolysis significantly alleviates brain injury. Quercetin, a natural flavonol compound, can significantly reduce diabetes-induced platelet aggregation (Mosawy et al., 2014), which may be related to the inhibition of compact platelet granule exocytosis (Mosawy et al., 2013b). Similarly, quercetin inhibits agonists (ADP, collagen, and thrombin) as well as induces platelet aggregation and granule secretion (Liang et al., 2015). Quercetin also binds to the GPIIb/IIIa platelet receptor, thus inhibiting the aggregation-promoting properties of calcium ion carriers and avoiding an increase in platelet-derived particles; these improve hemorheology (Zaragozá et al., 2021) and reduce thrombosis after carotid artery injury induced by FeCl3 in C57BL/6 rats (Mosawy et al., 2013a). Quercetin also effectively blocked *in vivo* FeCl3-induced arterial thrombosis and reduced IS infarct volume by inhibiting glycoprotein VI (GPVI)-mediated platelet signal transduction (Oh et al., 2021).

The binding of collagen to GPVI leads to receptor aggregation, which stimulates tyrosine phosphorylation and thus causes platelet aggregation (Gibbins et al., 1996; Poole et al., 1997). Quercetin inhibits platelet activation by inhibiting various components of the GPVI signaling pathway (e.g., collagenous tyrosine phosphorylation) (Hubbard et al., 2003; Hubbard et al., 2006; Wright et al., 2010), which may be a key factor for improving nerve injury in IS. In clinical trials, platelet aggregation was inhibited 30 and 120 min after oral quercetin administration, with a corresponding reduction in collagen-stimulated platelet tyrosine phosphorylation (Hubbard et al., 2004). Quercetin also inhibited platelet aggregation when collagen was stimulated at concentrations between 0.5 and 1.0 μg/ml, with IC50 values below 3 μM (Hubbard et al., 2003). Thus, these results support the clinical transformation of quercetin.

In contrast, Lee et al. (2013) found that quercetin did not reduce PT, aPTT, or platelet aggregation in experimental rats. They revealed that its downregulation of mitogen-activated protein kinase (MAPK) activation restricted tissue factor expression, thereby prolonging the time for atherothrombosis development. In endothelial cells, quercetin transcription induces the human t-PA gene by requiring a specific Sp1 (b) element within the proximal promoter region, which is mediated by the P38 MAPK-dependent signaling pathway (Pan et al., 2008).

Protein disulfide isomerase (PDI), a thiol isomerase secreted by vascular cells, is required for thrombosis. Quercetin-3-rutinoside prevents thrombosis in a PDI-dependent manner in experimental rats (Jasuja et al., 2012). Oral administration of 1,000 mg isoquercetin can reduce the plasma concentration of D-dimer by 21.9% and inhibit the activity of PDI in the plasma, thereby exerting an antithrombotic effect (Zwicker et al., 2019). This was related to the reduction of platelet-dependent thrombin content by blocking the production of platelet factor Va (Stopa et al., 2017). Intravenous administration of quercetin significantly attenuated TNF-α levels and prothrombin activity in a rabbit model of LPS-induced DIC (Yu et al., 2013) (Figure 2). Nonetheless, oral quercetin did not prevent thrombo-embolic stroke in an earlier Dutch cohort study (Knekt et al., 2000). This finding may be related to the use of dietary quercetin content as an intervening factor. Table 2 summarizes the role of quercetin and its derivatives in limiting thrombosis.

**TABLE 2:** Anti-platelet aggregation effect of quercetin.

Ingredient	Dose	Experimental Model	Mechanism	References
quercetin	6 mg/kg	C57BL/6 mice; FeCl3-induced carotid artery injury	↓GPIIb/IIIa activation; ↓platelet granule exocytosis; Inhibits platelet aggregation	Mosawy et al. (2013a)
quercetin-3-rutinoside	0.5 mg/kg	C57BL/6J mice; Laser-induced injury/FeCl3 injury	Inhibit fibrin production; ↓Expression of PDI; Inhibits platelet aggregation and thrombosis	Jasuja et al. (2012)
isoquercetin	500 mg or 1,000 mg	Healthy cohort (a-e) abstaining from quercetin-rich foods 72 h before intervention, excluding those with oral anticoagulants or antiplatelet drugs	↓PDI activity; ↓platelet factor Va; ↓platelet-dependent thrombin	Stopa et al. (2017)
quercetin	2 mg or 10 mg	SD rats, FeCl3-induced carotid artery injury	↓blood triglyceride, ↓glucose levels; ↓tissue factor, ↓MAPK activation	Brüll et al. (2015)
quercetin	_	Healthy non-smokers with normal coagulation function, not using immunologics, antiplatelet, NSAIDsetc.	Blocks GPIIb/IIIa receptors; ↓Platelet activation and aggregation	Zaragozá et al. (2021)
quercetin	0.5 mg/kg, 1 mg/kg, 2 mg/kg	Adult male New Zealand white rabbits, DIC experimental models	↑Protein C and ATIII; ↓APTT, ↓PT, ↓TNF-α; Anti-inflammatory and anticoagulant	Yu et al. (2013)
quercetin	50 mg/kg, 100 mg/kg	Wild-type (WT, C57BL/6 strain, 6–8 weeks old, 18–22 g BW) male mice, FeCl3-induced *in vivo* thrombosis	↓granule secre, ↓ROS; ↓platelet aggregation; tionInhibition of αIIbβ3 integrin and GPVI signaling;	Oh et al. (2021)
quercetin	1 μM	Quercetin pretreated human umbilical vein endothelial cells for 0–24 h	↑ t-PA gene expression; activate p38MAPK	Pan et al. (2008)
quercetin	6 mg/kg	Diabetes C57BL/6 mice; FeCl3-induced carotid artery injury	↓granule exocytosis; Inhibits platelet hyperaggregation and thrombosis	Mosawy et al. (2014)
quercetin	5 mg or 69 mg	A two-treatment, randomized, double-blind, crossover study	↓Syk tyrosine phosphorylation; Inhibits collagen stimulated platelet aggregation	Hubbard et al. (2006)
quercetin-4¢-O-b-D-glucoside	150 mg or 300 mg	Those who did not take aspirin and a low quercetin diet 14 days before the study	↓Syk tyrosine phosphorylation; Inhibition of GPVI signal transduction	Hubbard et al. (2004)
Isoquercetin	500 mg or 1000 mg	Patients with advanced cancer; A multicenter, multidose, open-label phase II clinical trial	↓D-dimer, ↓platelet dependent thrombin; Inhibition of PDI activity	Zwicker et al. (2019)

### 4.3 The Role of Histomorphology

**FIGURE 3:**
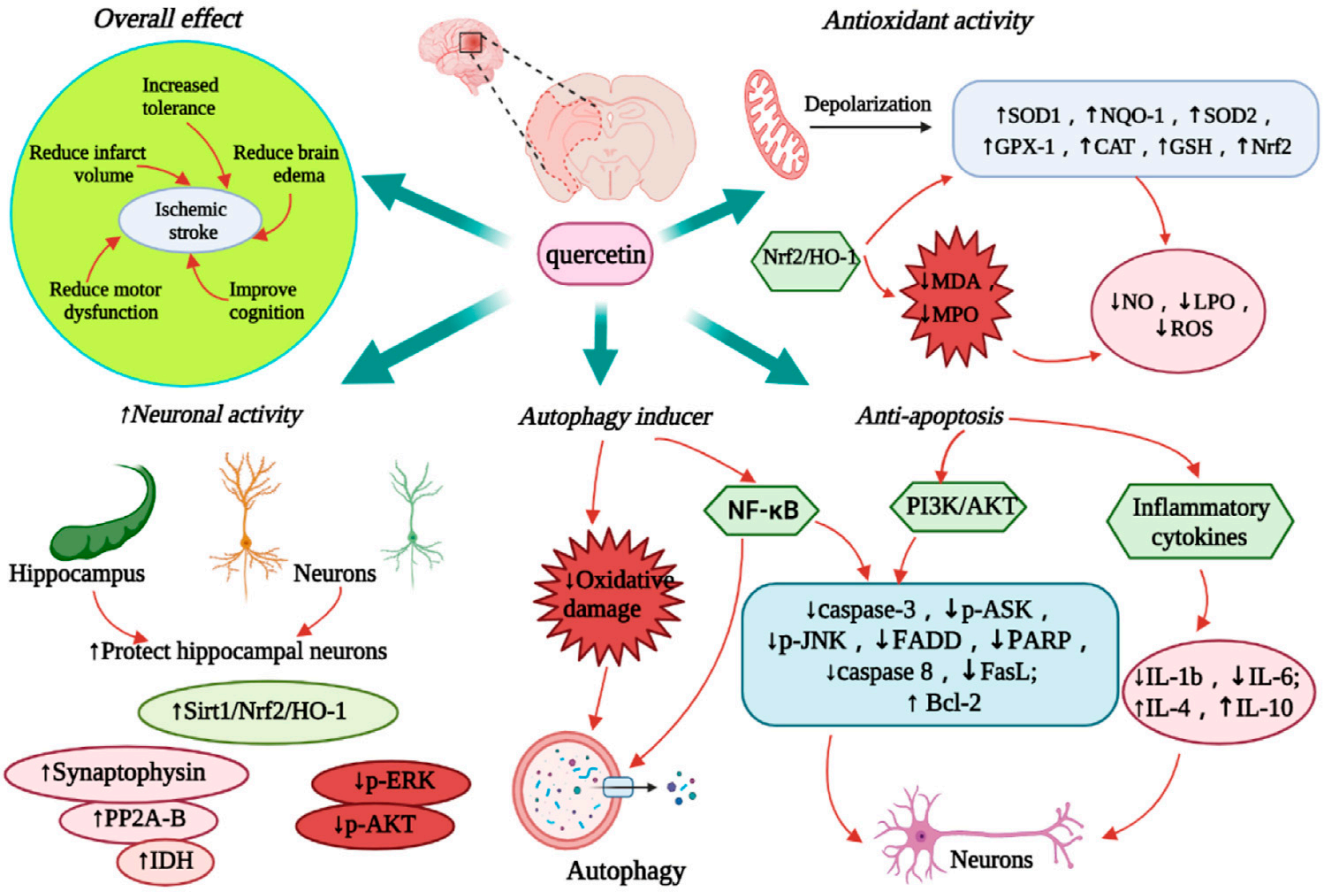
Diagram of the neuroprotective effect of quercetin on IS. The figure highlights the overall therapeutic effect of quercetin, anti-oxidative stress, anti-apoptosis, promoting autophagy and neuroprotective activity. Abbreviations: ↑, increase; ↓, decrease; SOD, Superoxide dismutase; NQO-1, NAD(P)H dehydrogenase quinone-1; GPX-1, glutathione peroxidase-1; CAT, catalase; NOS1, nitric oxide synthase 1; GSH, glutathione; Nrf2, nuclear factor 2-related factor 2; HO-1, Heme oxygenase-1; NO, nitric oxide; LPO, lipid peroxidation; ROS, reactive oxygen species; IL, interleukin; Sirt1, sirtuin 1; NF-kB, nuclear factor kappa B; IDH, isocitrate dehydrogenase; PP2A, Protein phosphatase 2A; FADD, Fas-associated death domain; PARP, poly (ADP-ribose) polymerase; PI3K, phosphoinositide 3-kinase; AKT, protein kinase B; ASK, apoptosis signal-regulating kinase; JNK, c-Jun N-terminal protein kinases; ERK, extracellular-signal-regulated kinase; FasL, factor associated suicide ligand.

In an *in vitro* ischemia model, quercetin-treated cells showed improved tolerance to oxygen-glucose deprivation (OGD) or oxygen glucose recovery (ROG) (Lee et al., 2016). Quercetin administration reduced the corrected total infarct volume and edema percentage by 43.6% and 48.5%, respectively, along with a significant behavioral recovery effect (Lee et al., 2015). Quercetin and Kolaviron pre-treatment significantly improved the I/R-induced changes in brain water content. Significant remission of cerebral infarction was observed in the Kolaviron and quercetin treatment groups (Akinmoladun et al., 2015), which might be linked to the role of quercetin in the Sirt1/nuclear factor-erythroid 2-related factor 2 (Nrf2)/heme oxygenase-1 (HO-1) signaling cascade (Yang et al., 2021). Compared to free quercetin or quercetin-carrying exosome (quercetin-EXO) therapy, treatment with quercetin/mAb gAP43-EXO dramatically reduced infarct size and improved neurological recovery in MCAO reperfusion-induced rats (Guo et al., 2021) (Figure 3). Simultaneously, quercetin improved the IS-associated motor and sensory deficits in the dorsal striatum, which may be related to the upregulation of MC4R-mRNA expression (Ulya et al., 2021). More intuitively, quercetin showed 6.79 2 ± 0.41 right turn in rats in the permanent MCAO model and 9.31 ± 0.33 right turn in the control group. Most rats in the treatment group showed mild to moderate neuromotor deficits (*p* < 0.0001) (Ahn and Jeon, 2015). In addition, the infarct volume of rats in the control and treatment groups was 26.35 ± 2.25% and 14.87 ± 1.75%, respectively (Park et al., 2020). Quercetin can improve cognitive function in rats with ischemic injury. In the Morris water maze (MWM) test, quercetin therapy restored spatial learning deficits by increasing the time and amount of access to the central region (Le et al., 2020). By boosting the number of new Olig2+ oligodendrocyte progenitors in the subventricular zone, quercetin alleviated hypoxia/ischemia (HI)-induced cognitive impairment (Qu et al., 2014). Compared with the control group, I/R rats pre-treated with quercetin (20 mg/kg) for 7 days showed a significant reduction in cognitive impairment as well as improvement in motor capacity, cerebral edema, and infarct volume (*p* < 0.001) (Viswanatha et al., 2018; Viswanatha et al., 2019). Table 3 summarizes the neuroprotective effects of quercetin on IS.

**TABLE 3:** Neuroprotective effects of quercetin in different IS models.

Ischemic stroke	*In vitro*/*In vivo*	Dose	Effective Molecular Mechanism	References
MCAO	*in vitro*/*in vivo*	10 mg/kg	↓ PP2A subunit B; inhibition of glutamate toxicity	Park et al. (2019)
MCAO/R	*in vitro*/*in vivo*	3.4 mg/ml	↑NQO-1, ↑HO-1, ↑SOD1, ↑GPx1; ↓ROS activation of Nrf2/HO-1 pathway; reduce I/R damage;	Guo et al. (2021)
OGD	*in vitro*	10 µM	↑synaptophysin; promote the growth of neurites	Orbán-Gyapai et al. (2014)
MCAO/R	*in vivo*	30 mg/kg	↑GPx, ↑SOD, ↑CAT; ↓PARP, ↓caspase-3, ↓p53, ↓LPO; protection of Na_+_K_+-_ATPase Activity	Ahmad et al. (2011)
MCAO	*in vivo*	30 mg/kg	↓caspase-3, ↓PARP; inhibit the apoptosis pathway; reduce neuronal defects and neuronal degeneration	Park et al. (2018)
Focal cortical ischemia	*in vivo*	25 μmol/kg	↓MMP-9; reduce the damage of BBB	Lee et al. (2011)
pMCAO/Glutamate	*in vitro*/*in vivo*	10 mg/kg	↑Bcl2; ↓caspase-3, ↓Bax; reduce calcium overload of intracellular and hippocampal neurons	Park et al. (2020)
HIBI/OGD	*in vitro*/*in vivo*	50 mg/kg	↓IL-1β, ↓IL-6, ↓TNF-α; ↑SOD1,↑ SOD2, ↑GPX-1, ↑CAT; increase cell viability; Inhibit TLR4/NF-κB signaling pathway; improve dyskinesia and cognitive impairment	Le et al. (2020)
pMCAO	*in vivo*	30 mg/kg	↑ GSH; protect neurons and glial cells	Rivera et al. (2008)
MCAO	*in vivo*	10 mg/kg	↑[NAD+], ↑adenosine homocysteinase, ↑pyruvate kinase, ↑carboxy terminal hydrolase L1; ↓HSP60, ↓HSP2	Shah et al. (2018)
MCAO/HUMSCs	*in vivo*	25 mol/kg	↓caspase-3, ↓IL-6, ↓IL-1b; ↑IL-4, ↑IL-10, ↑transforming growth factor-b1; promote nerve function recovery	Zhang et al. (2016)
MCAO	*in vivo*	10 mg/kg	reduce infarct size and edema; antioxidant and neuroprotective activity	Lee et al. (2015)
MCAO/OGDR	*in vitro*/*in vivo*	25 mg/kg	anti-oxidative, anti-inflammatory, and antiapoptotic effects; reduces changes in ERK/Akt phosphorylation and protein phosphatase activity	Wang et al. (2020)
common carotid artery occlusion and reperfusion	*in vivo*	2.7 mg/kg	↓iNOS,↓ caspase-3, ↓ROS; ↑ HO-1, ↑SOD1, ↑GSH; protect the mitochondrial membrane; protect mitochondrial membranes and neuronal cells	Ghosh et al. (2013)

### 4.4 Prevention of Oxidative Stress

#### 4.4.1 Quercetin

The mitochondria are the main source of oxidative stress. Quercetin can activate mitochondrial large-conductance Ca^2+^ to regulate potassium (mitoBKCa) channels, participate in mitochondrial depolarization, and protect brain tissue from HI damage (Kampa et al., 2021). Quercetin synergistically enhances mitochondrial spare respiration, maintains neuronal mitochondrial function, and increases the expression of CREB target genes (PGC-1a), which promote neuronal survival and mitochondrial biogenesis in an OGD model (Nichols et al., 2015). Furthermore, quercetin can control the Sirt1/Nrf2/HO-1 pathway, thus drastically reducing ROS formation following IS (Yang et al., 2021). In several IS models, quercetin revealed a dose-dependent reversal of OGD-induced declines in superoxide dismutase-1 (SOD1), SOD2, glutathione peroxidase-1 (GPX-1), and catalase (CAT) levels (Le et al., 2020), which may be achieved by its LPO-reducing capability (Shalavadi et al., 2020). Another study pointed out that quercetin also regulates the expression of oxidase and other antioxidant enzyme genes, thereby preventing IS-associated oxidative stress (Yamagata, 2019). Further studies showed that quercetin induced the expression of Nrf2 in erythrocytes, thus strongly inhibiting the production of adhesion molecules; this action may be related to the antioxidant effect of HO-1 (Li C. et al., 2016). Lee et al. (2016) discovered that quercetin increased the expression of Nrf2, HO-1, and nitric oxide synthase 1 (NOS1) in SHSY5Y cells, thus indicating its antioxidative stress impact.

#### 4.4.2 Quercetin and Other Herbs

Quercetin and other herbal pre-treatments inhibit I/R-induced decreases in catalase and SOD enzyme activity, prevent LPO production, and increase GSH levels (Viswanatha et al., 2018; Viswanatha et al., 2019). Additionally, reduced NO and hippocampal lactate dehydrogenase (LDH) levels were observed in the cortex, striatum, and hippocampus of I/R rats (Ojo et al., 2019). Furthermore, intragastric injection of quercetin and rutin 10 min before reperfusion significantly reduced malondialdehyde (MDA) and myeloperoxidase (MPO) levels, increased endogenous antioxidant enzyme SOD and CAT levels, and improved I/R-induced inflammatory response (Annapurna et al., 2013).

#### 4.4.3 Optimization of Quercetin

Optimization of quercetin can significantly improve its efficiency and pharmacological effects across the BBB. Quercetin liposome preparations slow down the decline of GSH levels in the ipsilateral striatum and cortex after ischemia; it also maintains GSH levels in the ischemic areas and increases GSH concentration in neuronal and glial cells (Rivera et al., 2008). During cerebral I/R, intracellular GSH levels significantly increased in young and old rats receiving nano-quercetin (27 mg/kg) (Ghosh et al., 2013). Quercetin/mAb GAP43-Exo targets neurons by mediating mAb GAP43, thus enhancing the accumulation of quercetin in the ischemic areas as well as inhibiting ROS production by activating the Nrf2/HO-1 pathway to increase LDH levels (Guo et al., 2021). Quercetin/mAb GAP43-Exo decreased oxidative stress-induced I/R damage by boosting the nuclear translocation of Nrf2 and upregulating the transcription of NAD(P)H dehydrogenase quinone-1 (NQO-1), HO-1, SOD1 and GPx1 (Guo et al., 2021) (Figure 3).

### 4.5 Protection of Hippocampal Neurons

IS significantly induced endogenous neurogenesis in the dentate gyrus of the hippocampus. However, newborn neurons are difficult to differentiate into mature neurons (Arvidsson et al., 2002; Doeppner et al., 2011). Quercetin maintains isocitrate dehydrogenase levels in MCAO animal models and helps to preserve neuronal cell energy production, thereby reducing IS-induced neuronal cell damage (Shah et al., 2018). Moreover, quercetin attenuates the decrease in PP2A subunit B expression caused by glutamate treatment, thus further reducing neuronal cell death (Park et al., 2019). Through the Sirt1/Nrf2/HO-1 signaling pathway, quercetin restores the normal structure of hippocampal neurons in I/R mice with severe neuronal injury (Yang et al., 2021). Quercetin also reduces the activity and pathophysiology of the following processes: protein tyrosine and serine/threonine phosphatase in rat cortical tissue, oxygen-glucose deprivation/reoxygenation (OGD/R) in hippocampal slices and neuronal/glial cell lines, phosphorylation of ERK and Akt, and I/R-induced hindbrain damage (Wang et al., 2020). Quercetin treatment can also significantly increase the activity of SHSY5Y cells and E18 mouse cortical neurons (Lee et al., 2016), enhance the expression of synaptophysin in PC12 cells in the OGD model, and promote neurite growth in PC12 cells (Orbán-Gyapai et al., 2014). Three days after reperfusion, oral administration of nano-encapsulated quercetin reduced the activity of iNOS and caspase-3, expanded the number of neurons in the hippocampus, and prevented neuronal cell damage (Ghosh et al., 2013) (Figure 3).

### 4.6 Promotion of Autophagy

The mechanism of quercetin-induced autophagy in cell survival is complex because of the large number of biomolecules involved in this process. Based on the scope of the damage caused by HI, autophagy is used as a pro-apoptotic signal, wherein quercetin can be used as its inducer (Costa et al., 2016). In models of oxidative damage and ischemia, studies have revealed that the protective impact of quercetin is directly linked to the induction of autophagy (Wu et al., 2017). As a result, autophagy is linked to the pro-survival mechanism of quercetin in IS-induced brain injury and other related events (Wang et al., 2011; Zhi et al., 2016; Granato et al., 2017; Liu et al., 2017) (Figure 3). Quercetin has a protective effect against MCAO-induced neuronal cell apoptosis and likewise induces autophagy-mediated neuronal PC12 cell survival (Ahn and Jeon, 2015; Park et al., 2018). In IS, if myeloid cells lack an autophagy response, inflammatory glial cells would thus play a significant role in neuronal cell apoptosis by increasing ischemia; this happens in compensation for the reduced activity of myeloid cells (Kotoda et al., 2018). In this case, autophagy protected the neurons from ischemia-induced cell death. Surprisingly, quercetin, like many other polyphenols, induces autophagy (Pallauf and Rimbach, 2013). Quercetin plays a role in cellular survival by activating autophagy in brain myeloid cells (Chang et al., 2017). Furthermore, in MCAO-induced ischemia, quercetin altered the apoptosis/autophagy interaction and its linkage with the nuclear factor kappa B (NF-κB) signaling pathway by upregulating ubiquitin carboxy-terminal hydrolase L1, which is a related gene enzyme, at almost double the rate (Chirumbolo et al., 2019).

### 4.7 Inhibition of Apoptosis

Quercetin upregulates the intracellular Ca^2+^ concentration in the cerebral cortical and hippocampal neurons of MCAO rats. It also regulates the gene expression of Bcl-2, Bax, and caspase-3, thereby preventing apoptosis (Park et al., 2020). Another study found that quercetin reduced HI-induced cortical cell death by blocking the neuro-inflammatory response mediated by the toll-like receptor 4 (TLR4)/nuclear factor-kappa B (NF-κB) signaling pathway (Wu et al., 2019). The anti-apoptotic activity of quercetin may be due to its ability to suppress inflammatory genes in BV2 microglia (Mrvová et al., 2015). In addition, quercetin has also been reported to improve I/R-induced cognitive deficits as well as inhibit neuronal apoptosis by increasing p-Akt and decreasing p-ASK1, P-JNK3, cleaved caspase-3, and FADD protein expressions (Pei et al., 2016). Furthermore, quercetin not only inhibits acid toxicity mediated by acid-sensing ion channels, but also improves neuronal apoptosis in focal cerebral ischemia by reducing caspase-3 and PARP expression through the PI3K/Akt pathway (Park et al., 2018). After local cerebral ischemia, human umbilical cord mesenchymal stem cells (HUMSCs) transplanted with quercetin can reduce pro-inflammatory cytokines IL-1B and IL-6, increase anti-inflammatory cytokines IL-4 and IL-10, inhibit the expression of apoptosis factor caspase-3, and promote the recovery of nerve function (Zhang et al., 2016). Furthermore, isorhamnetin (30-methoxy-3,40,5, 7-tetrahydroxy flavanone), a quercetin metabolite, has been demonstrated to lower blood pressure and endothelial dysfunction in spontaneously hypertensive rats (Sánchez et al., 2006; Sanchez et al., 2007). In the methylglyoxal-binding OGD model, isorhamnetin inhibited caspase 8 activation and decreased Fas and FasL expression, thereby lowering the activation and ability of NF-κB to perform an anti-mitochondrial-dependent apoptotic role (Li W. et al., 2016) (Figure 3).

## 5 Conclusion and Perspectives

Quercetin has a unique chemical structure and is widely found in our daily diet (e.g., vegetables and fruits), thus making it easy to obtain. Quercetin has showed respectable therapeutic effects on IS-induced models. It inhibits inflammatory thrombosis, reduces cerebral edema, infarct size, and oxidative stress, promotes autophagy and anti-apoptosis, and can be used as an adjuvant agent in the treatment of IS. Importantly, quercetin has been found to inhibit platelet activation and limit inflammatory thrombosis in both animal and clinical studies. The anti-inflammatory properties of quercetin are mediated by the regulation of the expression of various inflammatory factors. It also prevents neuronal death by stimulating the NF-κB signaling pathway, which suppresses caspase-3 and Bax, and promotes Bcl-2 expression.

To date, a number of studies have suggested that quercetin can be used as a neuroprotective drug in the treatment of IS. However, owing to its poor bioavailability and ability to cross the BBB, its application in the clinical setting is limited. Therefore, future research should focus on optimizing the conformation of quercetin or developing a quercetin nano-drug delivery system to improve its bioavailability and BBB passing rate. Meanwhile, we should pay attention to the effects of quercetin on neurogenesis and synaptic plasticity after IS*.* More clinical trials should also be designed to clarify the effective dose of quercetin in the treatment of IS. Quercetin also has a variety of metabolic components in the body; only a few studies have focused on the pharmacological effects of its metabolites, thus warranting further research on its biochemical and metabolic properties.
